# Women's needs in their journey towards motherhood via oocyte donation: A mixed methods systematic review

**DOI:** 10.18502/ijrm.v21i6.13633

**Published:** 2023-07-24

**Authors:** Hamideh Jafari, Ali Taghipour, Hosein Ebrahimipour, Robab Latifnejad Roudsari

**Affiliations:** ^1^Student Research Committee, Mashhad University of Medical Sciences, Mashhad, Iran.; ^2^Social Determinants of Health Research Centre, Mashhad University of Medical Sciences, Mashhad, Iran.; ^3^Department of Epidemiology, School of Health, Mashhad University of Medical Sciences, Mashhad, Iran.; ^4^Social Determinants of Health Research Centre, Mashhad University of Medical Sciences, Mashhad, Iran.; ^5^Nursing and Midwifery Care Research Center, Mashhad University of Medical Sciences, Mashhad, Iran.; ^6^Department of Midwifery, School of Nursing and Midwifery, Mashhad University of Medical Sciences, Mashhad, Iran.

**Keywords:** Need, Women, Oocyte donation, Motherhood.

## Abstract

**Background:**

Given the significant changes in family formation through donation procedures, providing an optimal level of care that is responsive to the needs of mothers who get pregnant via oocyte donation is pivotal to improve their maternal role. Therefore, it is necessary to recognize the needs of oocyte donation mothers to address their specific needs.

**Objective:**

This study aimed to review the needs of women in their journey towards motherhood via oocyte donation.

**Materials and Methods:**

In this systematic review, which followed the updated Joanna Briggs Institute's methodological guidance for conducting a mixed methods systematic review, the quantitative observational and qualitative studies were searched through databases including PubMed, Web of Science, PsycINFO, the Cochran Library, and Google Scholar search engine. Letters to the editor, commentaries, magazine articles, articles without full text and abstracts presented in congresses were excluded. All English-language articles related to the needs of oocyte donation mothers, without time limitation, were reviewed. The eligible studies were critically appraised independently by 2 researchers.

**Results:**

4649 records were identified from those 18 articles were finally included in the review. The needs of oocyte donation mothers comprised 8 categories: The need for special services in fertility clinics, the need to improve the quality of care, the need for emotional support and psychological consultation, information needs, the need for financial support, the need for disclosure counseling, educational needs, and the need for sociocultural and religious support.

**Conclusion:**

This review suggests various needs of oocyte donation mothers. The results can be used in carefully planning supportive programs for this vulnerable population.

## 1. Introduction

Along with a rise in delaying parenthood and an increase in seeking assisted conception, oocyte donation as the last treatment is increasingly leading to motherhood for millions worldwide (1-3). Nearly 10% of all assisted reproductive technology cycles in the United States, with live birth rates upward of 50% per cycle, occur with oocyte donation amounting to 21,182 in 2015 (1). Nevertheless, for parents of children conceived with an oocyte donation, the psychological and emotional consequences of infertility and years of distress are not easily obliterated (4). Becker et al. stated that “parents of children conceived through an oocyte donation contest normative definitions of kinship and family, including stigma and otherness, resist challenges to the family they have built and, to some extent, rework their allegiance to cultural norms to suit their own needs" (5). It has been reported that some mothers are conflicted about their maternal role and did not feel like the mothers who had their child until later in the first year. It has also been hypothesized that mothers, over a longer period of time, will have to put in more emotional effort to understand the child as their own (2).

Given the challenges of parenthood following oocyte donation and ambivalence over the transition to a pregnant identity, these families may benefit from professional support to manage their issues and adjust to this transition (6). Positive pregnancy and childbirth experiences depend partly on receiving services from experienced counselors, realizing donation mother's expectations, and a well-functioning oocyte donation program (7-9). Proper preparation and careful planning for parenthood and satisfaction with successful treatment can decrease mental health problems in oocyte-donation mothers (10).

The fact is that with the extensive changes in family formation through oocyte donation, it is considered necessary to revise the psychosocial and lifelong needs of these women from pre-treatment onward (11). Providing an optimum level of care and services that are responsive to the needs of these mothers is key to improving their maternal role. Further studies are needed to recognize the facilitating and constraining factors in healthcare. Awareness of the needs of women who finally get pregnant after fertility treatment is necessary for midwives and healthcare professionals (12). Yet, no research has comprehensively and systematically investigated the needs of oocyte donation mothers during pregnancy, birth, and motherhood. Also, there is no national guideline in this regard (13). So, a wide-ranging review is needed to collect the available data and make the evidence accessible to provide the basis for tailoring appropriate support programs for oocyte donation mothers.

The present study therefore aimed to address this issue and identify the needs of oocyte donation mothers by systematically identifying, appraising, summarizing and synthesizing the relevant literature to address the specific needs of this vulnerable population.

## 2. Materials and Methods

### Design 

This review is a mixed methods systematic review of qualitative and quantitative studies. This study followed the updated Joanna Briggs Institute methodological guidance for conducting a mixed methods systematic review by Stern et al., taking a convergent integrative approach to synthesis. A convergent integrative approach involves data transformation and allows reviewers to combine quantitative and qualitative data (14). The reporting of this systematic review was guided by the reporting guideline for meta-analyses of observational studies in epidemiology (MOOSE) (15).

### Data sources and search strategy 

The articles were extracted from databases including PubMed, ISI Web of Science, PsycINFO, the Cochran Library, and the Google Scholar search engine up to August 2022. In addition, the references cited in the retrieved articles were also searched, manually. The search was performed extensively using Boolean operators with 3 groups of keywords using MESH terms including “oocyte/egg/gamete donation”, “women/pregnant women/mothers/motherhood/parents", “need/supportive care", “healthcare", and “emotional health", in all possible combinations.

The patient, intervention, comparison, outcome, and study design (PICOS) model was used to help break down the searchable elements of the research question into (P) participants: women who get pregnant via oocyte donation; (I) intervention/exposure: getting pregnant via oocyte donations; (C) control group: not applicable; (O) outcomes: needs, wants, desires, expectations and preferences of oocyte donation mothers; and (S) study type: quantitative and qualitative studies.

### Study eligibility criteria

Eligibility criteria for the review included quantitative observational studies, including cross-sectional, case-control, and cohort studies, as well as qualitative studies with any design in English language related to the needs of oocyte donation mothers without time limitation. Duplicate and unrelated papers, articles without a full text, letters to the editor, commentaries, magazines articles and abstracts presented in congresses were excluded.

### Selection process

Titles and/or abstracts of retrieved studies using the search strategy and those from other sources were screened by 2 authors independently to potentially identify studies that met the eligibility criteria of this review. The full text articles were then assessed for eligibility independently by H.J and R.L.R.

### Data collection process

Firstly, it was decided to collect the data related to the study including author, year of publication, setting, design, sample, data collection tools, results, and quality score. The data were then extracted independently by 2 reviewers and entered into a checklist planned by the research team beforehand.

### Quality assessment

The eligible studies with the inclusion criteria were appraised independently by 2 reviewers, using strengthening the reporting of observational studies in epidemiology STROBE statement (16). Also, qualitative studies were assessed based on the Joanna Briggs Institute critical appraisal checklist for qualitative research (17). The researchers discussed any disagreements to resolve them. Finally, 3 studies were excluded due to poor quality. Other studies were evaluated as moderate and good and had adequate quality to enter the study.

### The method of synthesis 

To integrate qualitative and quantitative data in a way that fully informs the topic, one approach is transforming data into a mutually compatible format. Data transformation was carried out by converting quantitative data into qualitative data (i.e., qualitizing), which refers to quantitative data being converted into themes, categories, or narratives. In this review, the various needs of mothers who got pregnant via oocyte donation reported in different studies were grouped. The extracted data were then converted to different categories.

### Ethical considerations

This study was approved by Mashhad University of Medical Sciences, Mashhad, Iran under code of 990842. It is also approved by university Local Research Ethics Committee (Code: IR.MUMS.REC.1399.405).

## 3. Results

**Figure 1:**
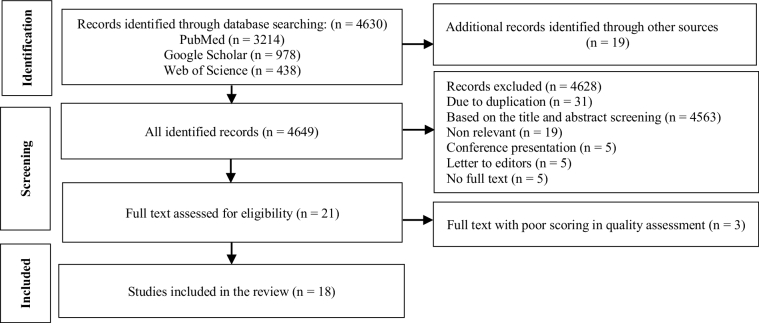
MOOSE diagram for the selection process of the articles.

In this study, a total of 4649 records were identified. After screening and quality assessment, 18 articles were finally included in the review to explore the needs of women who get pregnant via oocyte donation (Figure 1). The articles were published from 1997-2021 in different countries, including 4 studies conducted in the United Kingdom (2, 18-20), 5 in the United States (4, 5, 21-23), 5 studies in Iran (24-28), 2 studies in the Netherlands (12, 29), 1 study in Belgium (30) and 1 study in Sweden (31).

Out of 18 studies, the design of 7 studies was observational and 11 studies were conducted using qualitative approaches. In total, 8 categories of needs were extracted: the need to special services in fertility clinics (4 studies), the need to improve the quality of care (3 studies), the need for emotional support and psychological consultation (12 studies), information needs (6 studies), the need for financial support (2 studies); the need for counseling about disclosure (9 studies), the need for education and future research (5 studies), the need for sociocultural and religious support (7 studies) (Table I).

Previous experience with infertility treatment, years of distress, loss, and the “grief work" with infertility are not easily erased and may be ongoing after the child's arrival, discouraging parents from preparing for birth and parenthood. Still, it can be included in a functional and healthy sense of self, with attention to the needs of women from the beginning of treatment until after birth (4, 32). So, in this study, the needs of women during the entire process of accepting oocyte donation and treatment till after delivery were investigated.

**Table 1:** Characteristics of the included studies


**Author, Year (Ref)**	**Country**	**Sample size**	**Study design**	**The needs identified**
**Ahuja ** ***et al.,*** ** 1997 (20)**	UK	217	Survey	Need to provide special services in fertility clinics Need for emotional support and psychological consultation Information needs Need for financial support Need for counseling about disclosure Need for education and future research need for sociocultural support
**Becker ** ***et al.,*** ** 2005 (5) **	US	84	Qualitative study	Need for emotional support and psychological consultation Need for counseling about disclosure Need for sociocultural support
**Van Berkel ** ***et al.,*** ** 2007 (29) **	Netherlands	106	Descriptive-comparative study	Need for emotional support and psychological consultation Need for counseling about disclosure
**Hershberger, 2007 (21)**	US	8	Qualitative study	Need to provide special services in fertility clinics Need for emotional support and psychological consultation Need for counseling about disclosure
**Hershberger ** ***et al*** **., 2008 (23) **	US	8	Qualitative study	Need to improve the quality of care Information needs Need for education and future research
**Laruelle ** ***et al.*** **, 2011 (30)**	Belgium	135	Retrospective study	Need to provide special services in fertility clinics Need for counseling about disclosure
**Isaksson ** ***et al.*** **, 2012 (31) **	Sweden	107	Longitudinal cohort study	Need for counseling about disclosure
**Latifnejad Roudsari ** ***et al.*** **, 2013 (24)**	Iran	25	Descriptive-correlational study	Need for sociocultural and religious support
**Eyzadyar ** ***et al.*** **, 2014 (25)**	Iran	11	Qualitative study	Need for emotional support and psychological consultation Need for sociocultural and religious support
**Jafari ** ***et al.*** **, 2015 (26)**	Iran	25	Descriptive-comparative	Information needs Need for counseling about disclosure
**Bagheri-Lankarani ** ***et al.*** **, 2016 (27)**	Iran	12	Qualitative study	Need for emotional support and psychological consultation Information needs Need for sociocultural and religious support
**Applegarth ** ***et al.*** **, 2016 (4)**	US	54	Cross-sectional study	Need for emotional support and psychological consultation Need for counseling about disclosure
**Warmelink ** ***et al.*** **, 2016 (12)**	Netherlands	1	Qualitative study	Need to improve the quality of care Need for emotional support and psychological consultation Need for education and future research
**Scully ** ***et al.*** **, 2017 (19)**	UK	16	Qualitative study	Need for sociocultural and religious support Need for education and future research
**Hammond ** ***et al.*** **, 2018 (18)**	UK	18	Qualitative study	Need for emotional support and psychological consultation Need for education and future research
**Imrie ** ***et al*** **., 2020 (2)**	UK	85	Qualitative study	Need to provide special services in fertility clinics Need for financial support Need for emotional support and psychological consultation Information needs
**Hershberger ** ***et al.*** **, 2020 (22)**	US	6	Qualitative study	Need to improve the quality of care Need for emotional support and psychological consultation Information needs
**Ghlitch-Khani ** ***et al.*** **, 2021 (28)**	Iran	20	Qualitative study	Need for emotional support and psychological consultation Need for counseling about disclosure Need for sociocultural and religious support

### Need to special services in fertility clinics

4 studies highlighted special services in fertility clinics, including the availability of anonymous and known donors; establishing a centralized gamete donor registry; providing remunerated oocyte donation; and accessing the profiles of the oocyte donors (family, nationality, medical and obstetrical history; physical characteristics; personality traits; and responsiveness to drug protocol). In addition, the need for after-hours appointments for women who are employed or have other daytime commitments has been highlighted. It has been revealed that limitations and problems such as large number of applicants and the limited number of fertility treatment centers, as well as the long waiting lists for appointments, indicate the need to pay more attention to thinking of strategies and planning to solve these problems (2, 20, 21, 30).

### Need to improve the quality of care 

Another category of needs was the need to quality of care improvement, including attention to various aspects of antenatal care, pre-donation counseling, care provided by the fertility clinic staff, client-oriented care, and physicians' empathy (12). Also, other needs were promoting empowerment by encouraging women to engage in aspects of decision-making, promoting a sense of control over infertility treatment and obstetric care, and allocating enough time, especially in the first consultation. Open, clear, timely, and consistent communication and availability of healthcare professionals, and continuity of care to improve the experience of donation and also allowing discussion of any concerns that may arise after the donation were another relevant needs of women (23). Additionally, the importance of monitoring patient satisfaction as well as positive aspects of care (waiting times, information provision, emotional support), a high-level skills and ethics of healthcare providers through behaviors that express empathy and a deep understanding of the women's experiences were among the other expectations and desires of egg donation mothers (8, 21, 23). Warmelink et al. discussed mothers' viewpoints in relation to the importance of the role of the midwife in understanding the course of pregnancy, the need for early and frequent ultrasound scans and monitoring of fetal heart sounds, as well as the need for a shorter time between consultations and extra consultations (12). To identify disease risk and health promotion, Hershberger et al. commented that “new models of care must be used in which donors, recipient parents, children, and donor half-siblings can communicate about genetic information and lineage, as well as providing confidential support about the donor's experience, disclosure, and relationship issues” (22).

### Need for emotional support and psychological consultation 

Most of the concerns that these families face, raise the need for emotional support and psychological consultation in the fertility clinic, including having a high level of anxiety during pregnancy about the bond with the baby, the outcome of the pregnancy, and long-term effects after treatment, as well as concerns about physical appearance and future contact with a known oocyte donor (4, 20-22, 27, 29). Other concerns indicate the need for counseling services, including worrying about feelings of an identity crisis or misery, a sense of experience worse than death in the family, having negative thoughts and blaming the lack of genetic link, and feelings of disability (21, 25, 29). Another unfavorable feelings that suggests the need for counseling are decreased self-confidence in the face of disclosure, sacrificing for the rights of the spouse, the desire to have a normal pregnancy, and a sense of being different from other pregnant women, which necessitate helping the mother to be adapted to pregnancy and develop an attachment to the fetus. Also, the stressful treatment, fatigue, despair, difficulties, and confusion in treatment show the need for emotional support and psychological consultation (18, 25).

There are also concerns about the thoughts of children that they are second order, a strange feeling, and a continuation of sadness. For some mothers, envisioning the child as their own involves more emotional work over a longer period of time because some women reported that they do not feel the child is their own until later in the first year (2). Other psychological challenges, such as self-esteem destruction, depression, spiritual discouragement, and harmful coping mechanisms, like aggression and denial, show the need for emotional support and counseling (28). Finally, one study discussed the need for more psychosocial support in the postpartum period in order to create opportunities to further discuss feelings (12).

### Information needs 

These needs are about providing information about the process of treatment and well-researched data for parents about the donor's age, profession, physical appearance, hobbies, place of residence, characteristics, medical history, race, smoking, alcohol and narcotics use, predominantly on medical and genetic history, personality, and intelligence, details of donor screening and matching, and oocyte allocation (12, 22, 27). Other information needs include the need to provide visual aids, written information, and a guidance booklet describing the procedure in more detail, such as potential risks, side effects, and long-term consequences and finally, the need to share the experiences (20).

### Need for financial support 

The need for financial support for patients with lower incomes and decreasing fertility treatment costs with oocyte donation was reported in the studies by Ahuja et al. and Imrie and co-workers (2, 20).

### Need for disclosure counseling 

There is a need for disclosure counseling about the time of disclosing, how to tell the child, the child's reaction, future donor interaction with offspring, and ensuring that disclosure is kept private (4). Other issues include potential identity issues, the questions that the child may ask about his origin, ongoing uncertainties about a disclosure decision, as well as making disclosure to others and intense worry about others judging, social stigma, the necessity of planning to disclose or not, disagreement between partners about disclosure to the child, and the need for providing appropriate counseling for different recipients with different perspectives about the donor (5).

### Educational needs 

High knowledge and skill levels are required in fertility clinics (23). The educational needs that egg donation mothers focused on included public education and training healthcare professionals to improve their skills. This training could be on giving care, treating with respect, and devoting time to questions and guidance of the recipients (20). They highlighted the need of midwives to receive training on empowering women during their transition to parenthood (12). Also, there was the need to train for chaplains and provide suitable training in communication for physicians and empathy skills during college and throughout their professional careers. Mothers also emphasized that particularly, when using of donated oocytes is announced publicly, due to the sadness and shock this news causes, they need to be supported by trained healthcare professionals (18, 19). There is a little evidence of the experiences of women who get pregnant after fertility treatment (12). So, keeping up-to-date with emerging research is necessary, and physicians and mental health professionals need to gain more knowledge about caring, curative problems, and limitations these mothers confront with. They should also need to be trained to use the research findings to counsel and reassure the parents (32).

### Need for sociocultural and religious support 

Egg donation mothers need to sociocultural and religious support for lowering social pressure, gaining public acceptance, and reworking their allegiance to cultural norms to suit their own needs (5, 24, 25, 27, 28). Additionally, addressing the sociocultural factors and using a holistic care approach, considering the psychological, social, and spiritual needs of mothers, and resolving their religious restrictions, seemed necessary (24, 28). The stigma associated with non-genetic parenthood emphasizes the need for support and cultural development (32). Bagheri-Lankarani et al. referred to the focus on religious beliefs and impediments for oocyte donation mothers (27). There is a need to resolve difficulties in obtaining information about the official religious teachings, particular barriers for access, distinctive ethical challenges, and the chance to discuss the religious implications of clinical decision-making in the clinic or the religious settings (19).

## 4. Discussion

The present study aimed to review the needs of women who get pregnant via oocyte donation. The needs in these women comprised 8 categories including the need for special services in fertility clinics, the need to improve the quality of care, the need for emotional support and psychological consultation, information needs, the need for financial support, the need for disclosure counseling, educational needs, and the need for sociocultural and religious support.

The need for fertility clinic support services was one of the needs of women in this review. Women's experience and satisfaction are affected by clinic support services, such as providing consultation conditions to people far from the clinic and introducing a known donor because some infertile women cannot find a known donor (30, 33). In a qualitative study, the need to offer counseling via video conferencing for women far from the clinic was discussed.

The need to improve the quality of care and providing person-centered care by clinics was another result of this review (34). Donation mothers expected a normal pregnancy but need care different from normal midwifery practice (12). In Finnish healthcare system, donation mothers could discuss details of their treatment, pregnancy, and issues concerning the donor with the health providers several times before starting the process of treatment (10). It has been reported that the quality and performance of fertility clinics should be clarified for the mothers. This includes enough appointments with the physicians, receiving services from a qualified team of health professionals, getting benefit from continuity of care, the ability to contact the team in case of any problems, not approaching with canceled hospital appointments, and unlimited access to the medical records. Also, having the freedom to choose a medical professional, receiving feedback after being discussed with the team, and paying attention to the client's privacy was other important issues. It has also been mentioned that treating others with understanding, giving people a private room to ask their questions, and leaving difficult or sensitive topics to a planned assessment were other salient issues to improve the quality of care (35).

The need for emotional support and psychological counseling at the infertility clinic has been emphasized in all 18 studies reviewed. Evidence suggests that becoming pregnant after fertility treatment and oocyte donation, like a double-edged sword, comes with many complex psychosocial issues and difficulties in the transition to parenthood, leading to perinatal morbidity (13). The journey of these forgotten women during the process of achieving treatment and successful pregnancy is often overlooked. Egg donation mothers often suffer from grief, disappointment, and experience ambivalent feelings (2, 9, 12). In the study by Imrie and co-workers, one woman described the lack of genetic link as a bit heartbreaking and still as an struggle at times to feel whether her child is completely hers, instead of imagining the donor as the mother of her child (32). Maternal distress could have more negative impact on children who have been informed of their origins (36). A growing body of evidence demonstrates that this group of women has specific psychological issues, which undoubtedly increases the need to attention (13). Oocyte donation couples must see a psychologist at least once. They are allowed for treatment if there was no problem after the screening for psychological status (7). The anxiety and fear of pregnancy are affected by the health professionals' support in primary care (6). Also, introducing examples of non-genetic close relationships can convince the parents that genetic connections are not essential in forming loving relationships (2). Therefore, long-term psychological counseling, during and after the donation process, with a mental health professional in the fertility clinic before pregnancy may improve women's psychological status (7-9).

The need for information was another important needs of oocyte donation mothers in this review. Before becoming pregnant, women should receive information about the potential risks of pregnancy as well as the health of newborn at a fertility clinic (7). Additionally, several studies have shown that recipients are interested in knowing the donor's information about age, occupation, physical appearance, interests, position, characteristics, medical history, and/or genetics, race, personality, and intelligence of the donor, as well as details of donor selection and matching, and oocyte allocation (8, 10, 12, 20, 22, 33, 35).On the other hand, the need to ensure health information and develop adequate sources of information is important (37). Also, the similar experiences of the mothers' transition to parenthood could help to reduce the stigma of oocyte donation families. Therefore, it is necessary to provide conditions for peer-sharing experiences in fertility clinics (2).

The need for financial support was another issue mentioned in this study. Legislations relating to the oocyte donation varies throughout the world. In some countries, such as the USA and the UK, most patients have to pay for treatment, so it is difficult to treat patients with lower incomes. In Spain, the public health system covers the cost of maximum of 3 fertility treatment cycles with oocyte donation for women under 40 yr (34, 38). Therefore, considering the inflated costs of infertility treatment, paying attention to insurance coverage and financial support seems necessary.

Another issue was the need for counseling about disclosure. Research suggests different feelings about disclosing information surrounding conception for oocyte donation. For example, in France, oocyte donation is anonymous (39). One view is that the child has a right to know the nature of fertilization; whereas, the other view tends to keeping it confidential for protecting the child, mother, and father, to maintain healthy family relationships, and also lack of an acceptable reason for the disclosure. Other key factors leading to confidentiality are child welfare, religious or cultural reasons, embarrassment at not being able to conceive, fear that the offspring may be treated differently, and the perception that family members may not accept the child (8, 9, 26, 33, 40, 41). Given the controversy over whether to disclose or not, there seems to be a need to guide donor-oocyte recipient women about the developing mother-child relationship (8, 41). In a study conducted in Sweden, significant groups of mothers following gamete donation expressed a need for counseling about how and when to talk to their children about donor conception. The couples undergoing donation treatment might benefit from opportunities to discuss with healthcare professionals for disclosure to other people and offspring early in the treatment process (31).

Another category in this review was the educational needs of mothers, focusing on general education and training of healthcare professionals to upgrade their skills. Achieving healthy pregnancy requires high technical and ethical knowledge and skills (23). Warmelink et al. have indicated the importance of training midwives and other maternity care providers regarding this subject (12).

Increasing public awareness about oocyte donation could also change the community beliefs (9, 42). There is a need for a shift to a psycho-educational partnership approach, as well as psychosocial professionals, who are trained and well-informed about up-to-date research literature and peer support opportunities (11). The community needs the availability and evidence-based information on oocyte donation. On the other hand, oocyte banks or educational campaigns like those used to promote blood donations may increase public awareness of the need for oocyte donors. Also, educational programs regarding the necessity of gamete donation could have the central role in these campaigns (34).

Consistent with our findings, socio-cultural and religious support were other needs that were neglected. The possibility of conceiving through a third party has been interpreted to befit reproductive norms and practices in different cultures. So, sharing oocytes is a social issue, and the formation of families using it brings sociocultural challenges (20, 42, 43). Some women experience stigma related to the non-genetic parenthood and confront it as badly reacted to by family and friends (32, 42). The way of its management depends on the meaning and perception by both the individuals involved in the third party reproduction and the social context within which they live; this needs public acceptance (5, 11). The surroundings of pregnant women affect their physical and mental health, also their child's prenatal development. An aware community could eliminate infertile couples' concerns and help them to overcome their problems (9). Finally, considering the need of egg donation mothers' to religious support, it is needed that in addition to the infertility care team, which pays attention to all the individual, psychological, social, and spiritual needs of infertile couples, religious scholars should also try to provide religious support through clarifying religious rules to the infertile patients (24).

The limitation of this study was using only English language publications. Also, despite our thorough search process, we may have missed or inadvertently excluded some relevant articles. However, to the best of our knowledge, this is the first systematic review conducted regarding the needs of oocyte donation mothers. Also, using of a comprehensive search strategy, over a longer period, systematically using studies conducted with different designs with mixed methods systematic reviews is the strength of this review and indicates the validity of the study findings.

## 5. Conclusion

This study is the first systematic review of the needs of women in their journey toward motherhood via oocyte donation. This review suggests the needs of oocyte donation mothers including the need to provision of special services; improved quality of care; emotional support and psychological consultation; essential information; financial support, counseling about disclosure; education and finally sociocultural and religious support. The results of this study can be used in careful planning for motherhood with oocyte donation. Identification of this wide range of perceived needs by women, who got pregnant via oocyte donation, could provide the basis for careful planning of supportive programs for this vulnerable population.

##  Conflicts of Interest

The authors declare that there is no conflict of interest.

## References

[B1] Deomampo D (2019). Racialized commodities: Race and value in human egg donation. Med Anthropol.

[B2] Imrie S, Jadva V, Golombok S (2020). "Making the child mine": Mothers’ thoughts and feelings about the mother-infant relationship in egg donation families. J Fam Psychol.

[B3] Melnick AP, Rosenwaks Z (2018). Oocyte donation: Insights gleaned and future challenges. Int J Fertil Steril.

[B4] Applegarth LD, Kaufman NL, Josephs-Sohan M, Christos PJ, Rosenwaks Z (2016). Parental disclosure to offspring created with oocyte donation: Intentions versus reality. Hum Reprod.

[B5] Becker G, Butler A, Nachtigall RD (2005). Resemblance talk: A challenge for parents whose children were conceived with donor gametes in the US. Soc Sci Med.

[B6] French LR, Sharp DJ, Turner KM

[B7] Sälevaara M, Punamäki R-L, Poikkeus P, Flykt M, Tulppala M, Tiitinen A (2016). Fear and experience of childbirth among women who conceived with donated oocytes: A prospective cohort study. Acta Obstet Gynecol Scand.

[B8] Hershberger P (2004). Recipients of oocyte donation: An integrative review. J Obstet Gynecol Neonatal Nurs.

[B9] Hadizadeh-Talasaz F, Simbar M, Roudsari RL (2020). Exploring infertile couples’ decisions to disclose donor conception to the future child. Int J Fertil Steril.

[B10] Sälevaara M, Punamäki R-L, Unkila‐Kallio L, Vänskä M, Tulppala M, Tiitinen A (2018). The mental health of mothers and fathers during pregnancy and early parenthood after successful oocyte donation treatment: A nested case‐control study. Acta Obstet Gynecol Scand.

[B11] Crawshaw M, Daniels K (2019). Revisiting the use of 'counselling' as a means of preparing prospective parents to meet the emerging psychosocial needs of families that have used gamete donation. Fam Relat Soc.

[B12] Warmelink JC, Adema W, Pranger A, de Cock TP (2016). Client perspectives of midwifery care in the transition from subfertility to parenthood: A qualitative study in the Netherlands. J Psychosom Obstet Gynaecol.

[B13] Younger M, Hollins-Martin C, Choucri L (2015). Individualised care for women with assisted conception pregnancies and midwifery practice implications: An analysis of the existing research and current practice. Midwifery.

[B14] Stern C, Lizarondo L, Carrier J, Godfrey Ch, Rieger K, Salmond S, et al (2020). Methodological guidance for the conduct of mixed methods systematic reviews. JBI Evid Synth.

[B15] Stroup DF, Berlin JA, Morton SC, Olkin I, Williamson GD, Rennie D, et al (2000). Meta-analysis of observational studies in epidemiology: A proposal for reporting. JAMA.

[B16] Von Elm E, Altman DG, Egger M, Pocock SJ, Gøtzsche PC, Vandenbroucke JP, et al (2014). The strengthening the reporting of observational studies in epidemiology (STROBE) statement: Guidelines for reporting observational studies. Int J Surg.

[B17] Joanna Briggs Institute https://jbi.global/critical-appraisal-tools.

[B18] Hammond K (2018). The role of normative ideologies of motherhood in intended mothers’ experiences of egg donation in Canada. Anthropol Med.

[B19] Scully JL, Banks S, Song R, Haq J (2017). Experiences of faith group members using new reproductive and genetic technologies: A qualitative interview study. Hum Fertil.

[B20] Ahuja KK, Mostyn BJ, Simons EG (1997). Egg sharing and egg donation: Attitudes of British egg donors and recipients. Hum Reprod.

[B21] Hershberger PE (2007). Pregnant, donor oocyte recipient women describe their lived experience of establishing the "family lexicon". J Obstet Gynecol Neonatal Nurs.

[B22] Hershberger PE, Driessnack M, Kavanaugh K, Klock SC (2020). Emerging views of kinships created through oocyte donation. MCN Am J Matern Child Nurs.

[B23] Hershberger PE, Kavanaugh K (2008). Enhancing pregnant, donor oocyte recipient women’s health in the infertility clinic and beyond: A phenomenological investigation of caring behaviour. J Clin Nurs.

[B24] Latifnejad Roudsari R, Jafari H, Taghipour A, Khadem N, Ebrahimzdeh S (2013). [The association of religious beliefs in infertile couples’ attitude towards donation procedures and its selection as a therapeutic approach to infertility]. Iran J Obstet Gynecol Infertil.

[B25] Eyzadyar N, Ahmadnia Sh, Seyedmirzaei SM, Azin SA, Yazdani Safa M (2014). [To choose the oocyte donation as a way of becoming a mother (Phenomenological study of infertile women’s in Royan Institute)]. J Iran Soc Stud.

[B26] Jafari H, Latifnejad Roudsari R, Taghipour A, Khadem Ghaebi N, Ebrahim Zadeh S (2015). [Comparison of knowledge and attitude towards reproductive donation procedures between recipient and non-recipient infertile couples at Mashhad Infertility Center]. J Torbat Heydariyeh Univ Med Sci.

[B27] Bagheri-Lankarani N, Zarei F, Zandi M, Omani Samani R, Karimi M (2016). The experiences of women fertilized through egg donation during their treatment process. Evid Based Care J.

[B28] Ghelich-Khani Sh, Kazemi A, Fereidooni-Moghadam M, Alavi M (2021). Psycho-social experience of oocyte recipient women: A qualitative study. BMC Women's Health.

[B29] Van Berkel D, Candido A, Pijffers WH (2007). Becoming a mother by non-anonymous egg donation: Secrecy and the relationship between egg recipient, egg donor and egg donation child. J Psychosom Obstet Gynaecol.

[B30] Laruelle C, Place I, Demeestere I, Englert Y, Delbaere A (2011). Anonymity and secrecy options of recipient couples and donors, and ethnic origin influence in three types of oocyte donation. Hum Reprod.

[B31] Isaksson S, Sydsjö G, Skoog Svanberg A, Lampic C (2012). Disclosure behaviour and intentions among 111 couples following treatment with oocytes or sperm from identity-release donors: Follow-up at offspring age 1-4 years. Hum Reprod.

[B32] Imrie S, Golombok S (2018). Long-term outcomes of children conceived through egg donation and their parents: A review of the literature. Fertil Steril.

[B33] Bracewell-Milnes T, Saso S, Bora Sh, Ismail AM, Al-Memar M, Hamed AH, et al (2016). Investigating psychosocial attitudes, motivations and experiences of oocyte donors, recipients and egg sharers: A systematic review. Hum Reprod Update.

[B34] Hogan RG, Hammarberg K, Wang AY, Sullivan EA (2022). 'Battery hens' or 'nuggets of gold': A qualitative study on the barriers and enablers for altruistic egg donation. Hum Fertil.

[B35] van Empel IWH, Nelen WLDM, Tepe ET, van Laarhoven EAP, Verhaak ChM, Kremer JAM (2010). Weaknesses, strengths and needs in fertility care according to patients. Hum Reprod.

[B36] Golombok S, Blake L, Casey P, Roman G, Jadva V (2013). Children born through reproductive donation: A longitudinal study of psychological adjustment. J Child Psychol Psychiatry.

[B37] Carter JR, Gezinski L, Karandikar Sh (2012). A comprehensive review of reproductive egg donation web sites. J Consum Health Internet.

[B38] Marre D, San Román B, Guerra D (2018). On reproductive work in Spain: Transnational adoption, egg donation, surrogacy. Med Anthropol.

[B39] Cordier C, Ducrocq B, Fry J, Catteau-Jonard S (2020). Views of French oocyte donors at least 3 years after donation. Reprod BioMed Online.

[B40] García D, Bautista O, Venereo L, Coll O, Vassena R, Vernaeve V (2013). Training in empathic skills improves the patient-physician relationship during the first consultation in a fertility clinic. Fertil Steril.

[B41] Hadizadeh-Talasaz F, Latifnejad Roudsari R, Simbar M (2015). Decision for disclosure: The experiences of Iranian infertile couples undergoing assisted reproductive donation procedures. Hum Fertil.

[B42] Latifnejad Roudsari R, Jafari H, Taghipour A (2019). The relationship of sociocultural beliefs and infertile couples' attitude toward reproductive donation: A descriptive-correlational study. Int J Reprod BioMed.

[B43] Behjati Ardakani Z, Navabakhsh M, Tremayne S, Akhondi MM, Ranjbar F, Mohseni Tabrizi A (2021). The impact of third party reproduction on family and kinship. J Reprod Infertil.

